# Proximal femoral bionic nail—a novel internal fixation system for the treatment of femoral neck fractures: a finite element analysis

**DOI:** 10.3389/fbioe.2023.1297507

**Published:** 2023-12-05

**Authors:** Kai Ding, Yanbin Zhu, Yifan Zhang, Yonglong Li, Haicheng Wang, Jiaxing Li, Wei Chen, Qi Zhang, Yingze Zhang

**Affiliations:** ^1^ Hebei Orthopaedic Clinical Research Center, Department of Orthopaedic Surgery, Hebei Medical University Third Hospital, Shijiazhuang, China; ^2^ Key Laboratory of Biomechanics of Hebei Province, Orthopaedic Research Institute of Hebei Province, Shijiazhuang, Hebei, China; ^3^ NHC Key Laboratory of Intelligent Orthopeadic Equipment, Hebei Medical University Third Hospital, Shijiazhuang, China; ^4^ Engineering Research Center of Orthopaedic Minimally Invasive Intelligent Equipment, Ministry of Education, Shijiazhuang, China; ^5^ Chinese Academy of Engineering, Bejing, China

**Keywords:** PFBN, CS, DHS, finite element analysis, tension force

## Abstract

**Introduction:** Currently, cannulated screws (CSs) and dynamic hip screws (DHSs) are widely used for the treatment of femoral neck fractures, but the postoperative complications associated with these internal fixations remain high. In response to this challenge, our team proposes a new approach involving triangular-supported fixation and the development of the proximal femoral bionic nail (PFBN). The primary objective of this study is to investigate the biomechanical differences among CSs, DHSs, and the PFBN in their capacity to stabilize femoral neck fractures.

**Methods:** A normal proximal femur model was constructed according to the CT data of a normal healthy adult. A femoral neck fracture model was constructed and fixed with CSs, DHSs, and the PFBN to simulate the fracture fixation model. Abaqus 6.14 software was used to compare the biomechanical characters of the three fracture fixation models.

**Results:** The maximum stresses and displacements of the normal proximal femur were 45.35 MPa and 2.83 mm, respectively. Under axial loading, the PFBN was more effective than DHSs and CSs in improving the stress concentration of the internal fixation and reducing the peak values of von Mises stress, maximum principal stress, and minimum principal stress. The PFBN fixation model exhibits superior overall and fracture section stability in comparison to both the DHS fixation model and the CS fixation model under axial loading. Notably, the maximum stress and peak displacement of the PFBN and bone were lower than those of the DHS and CS fixation models under bending and torsional loading.

**Conclusion:** The PFBN shows considerable improvement in reducing stress concentration, propagating stress, and enhancing the overall stability in the femoral neck fracture fixation model compared to DHSs and CSs. These enhancements more closely correspond to the tissue structure and biomechanical characteristics of the proximal femur, demonstrating that the PFBN has great potential for therapeutic purposes in treating femoral neck fractures.

## Introduction

With the aging of the population, cases of femoral neck fractures which occur between the femoral head and basal femoral neck are expected to reach 3 million by 2050, accounting for approximately 3.1% of the total fractures (Gullberg et al., 1997; Chen et al., 2017b). Literature reviews have shown that the mortality rate for femoral neck fractures can be as high as 20% within 1 year (Brauer et al., 2009; Klop et al., 2014). The primary surgical methods for treating femoral neck fractures include closed reduction with cannulated screws (CSs) and open reduction with dynamic hip screws (DHSs) (Sheehan et al., 2015). However, these treatment options are often associated with high rate of postoperative complications, and femoral head necrosis (up to 45%), shortening of femoral neck (up to 30%), and non-union (up to 19%) were the most common postoperative complications (Lu-Yao et al., 1994; Zlowodzki et al., 2008; Slobogean et al., 2015; Chen et al., 2017a).

The unique biomechanics comes from the special anatomy of the proximal femur, where joint forces are transformed into compression and tension forces through a significant bending moment and conveyed through the trabecular system in compression and tension (Aminian et al., 2007; Stiehl et al., 2007). The design concepts of CSs and DHSs are inherently incongruent with the anatomical structure and mechanics of the proximal femur, resulting in instability and stress concentration, leading to a high incidence of postoperative complications (Aminian et al., 2007; Johnson et al., 2017; Tianye et al., 2019; Fan et al., 2021; Xu et al., 2022). Furthermore, an attempt has also been made to increase the anti-compression force by adding additional screws and medial femoral support plates based on CSs and DHSs (Mir and Collinge, 2015; Zhuang et al., 2019; Li et al., 2023). However, these improvements increase tissue damage and overlook the design for anti-tension force, resulting in stress distribution, plate fractures, and limiting clinical application (Teng et al., 2022; Huang et al., 2023; Nan et al., 2023). Therefore, our team was the first to propose the concept of triangular-supported fixation (TSF) and design the proximal femoral bionic nail (PFBN) based on the triangular structure and mechanics characters of the proximal femur (Ding et al., 2022b; Xu et al., 2022). The key to TSF lies in the addition of support screws, creating a crossover structure that mimics the compression and tension trabeculae of the proximal femur, thus improving stress distribution and stability, which has been shown to be effective in intertrochanteric fractures (Ding et al., 2022a; Wang et al., 2022a; Wang et al., 2022b; Zhao et al., 2023).

The primary objective of this study was to analyze the biomechanical behaviors of the PFBN, DHS, and CS using finite element analysis to fix femoral neck fractures. It is hypothesized that the PFBN improves stress concentration and stress transmission in fracture fixation compared to the DHS and CS, thereby enhancing biomechanics and the clinical prognosis for treating femoral neck fractures.

## Materials and methods

This study was approved by the Ethics Committee of Hebei Medical University Third Hospital. Informed consent was signed by the volunteers.

### Constructing the proximal femur model

A healthy adult male weighing 60 kg and measuring 173 cm in height was selected to create a proximal femur model. A Siemens 64-row CT was used to scan the full-length femur (layer thickness: 0.625 mm), and the images were saved as DICOM format. The data were imported into Mimics21 to construct a three-dimensional model using thin multiplanar and volumetric reconstruction. Geomagic 2013 (Geomagic Company, United States) was utilized to generate the non-uniform rational basis spline surface.

### Establishing the fracture fixation model

**FIGURE 1:**
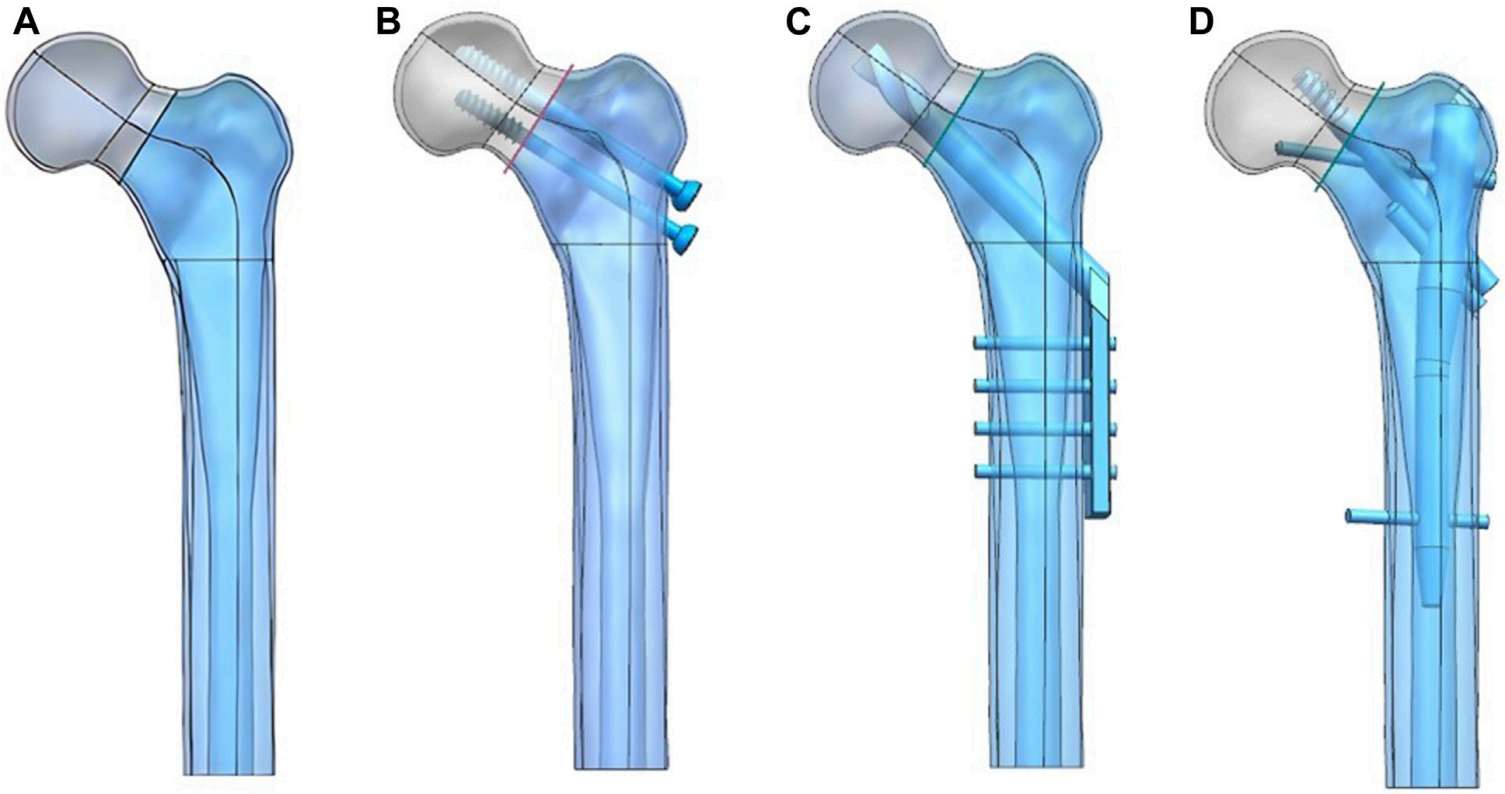
Femoral neck fracture model **(A)** was constructed and fixed with the CS **(B)**, DHS **(C)**, and PFBN **(D)**.

UG-NX 9.0 (Siemens Software, United States) was used to construct the Pauwels type III femoral neck fracture model and three internal fixation models, and the fracture fixation model was simulated according to the internal fixation placement (Figure 1). The C3D4 mesh model was constructed using HyperMesh 2013 (Altair Company, United States) software and imported into abaqus 6.14 (Dassault company, United States).

### Material properties and boundary loads

**FIGURE 2:**
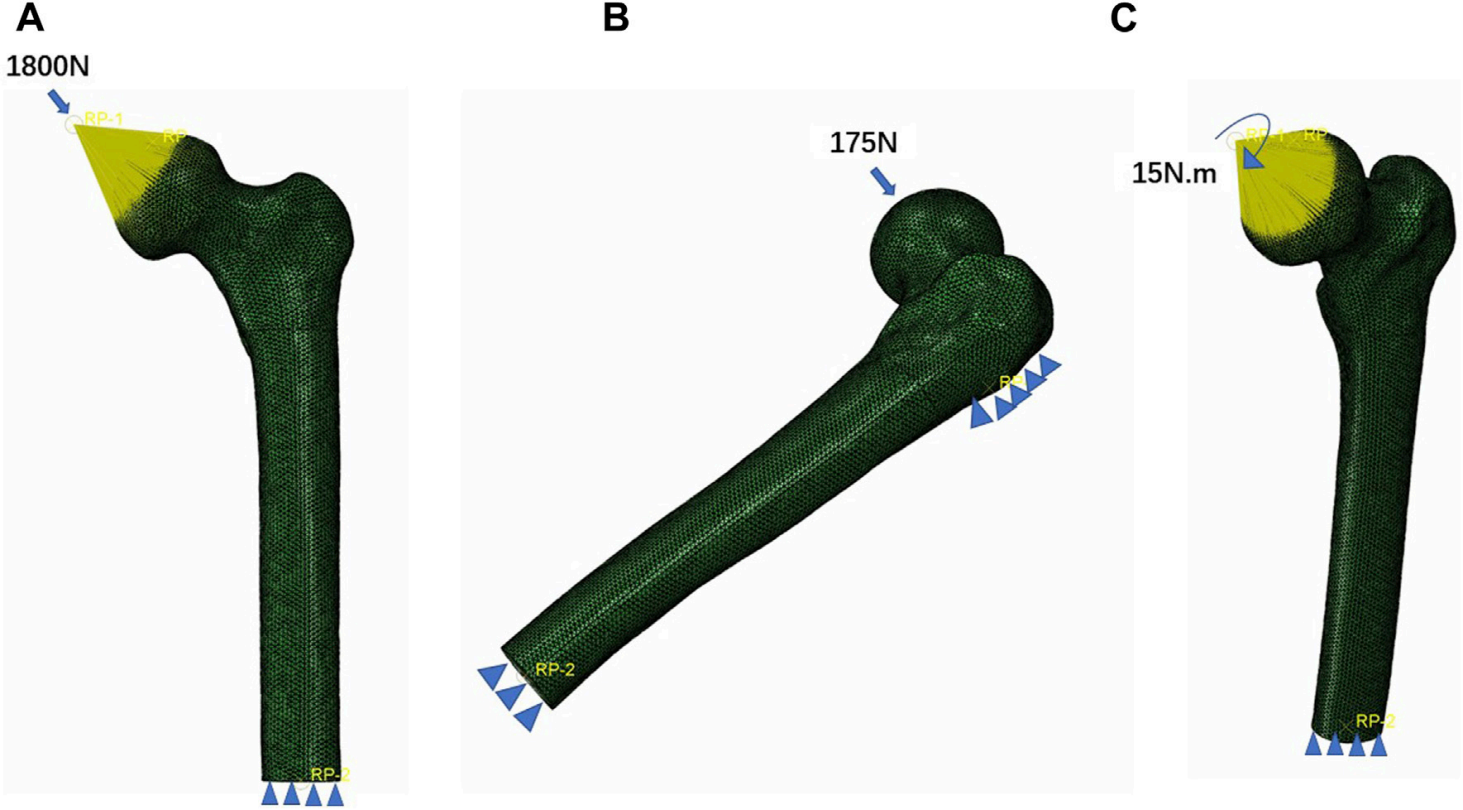
Three boundary conditions of the femoral neck fracture fixation models. **(A)** Axial load, **(B)** bending load, and **(C)** torsion load.

All bone and implant models were defined with isotropic and linear elastic properties. Based on prior literature (Ding et al., 2021a; Ding et al., 2021b; Ding et al., 2022a), the elastic moduli for cortical bone and cancellous bone were set to 17,000 and 1,500 MPa, respectively, with a Poisson’s ratio of 0.3 for both. The internal fixations were made of Ti6Al4V, with elastic moduli and Poisson’s ratios set to 110,000 MPa and 0.316, respectively (Sowmianarayanan et al., 2008). For the PFBN model, we assumed full bonding between the fixation screw, support screw, and the main nail. In the DHS model, the screw was tied to the main plate to mimic the holding force. We used tied interfaces to represent the screw thread/bone and screw/cortical bone interactions. The nail threads and partial nail body were set in binding relationships with the cancellous bone and cortical bone, respectively. All other bone–screw and bone–bone interfaces were defined as contact relationships. The coefficient of friction was set to 0.46 (Eberle et al., 2010; Zhan et al., 2020; Li et al., 2021). A load of 1,800 N (three times body weight load) is applied to the femoral head which was abducted 10°, tilted backward by 9° to simulate the one-leg standing, which is the maximum load on the hip joint during human walking (Li et al., 2019). A 15 Nm torsion load was applied to the surface of the femoral head along the axis of the femoral neck, representing the maximum load experienced during normal human gait (Huang et al., 2023). To ensure stability, the distal femur was fixed in all degrees of freedom. Additionally, a lateral load of 175 N was applied to the femur from the front, simulating a four-point load for bending (Huang et al., 2023) (Figure 2).

## Model validation

### Mesh convergence test

To ensure the accuracy of our analysis, the maximum stress of the proximal femur was used to analyze mesh convergence. We compared the maximum von Mises stress of normal proximal femur models with five element size meshes (3, 2.5, 2, 1.5, and 1 mm) and found that the maximum stress of the proximal femur model under the 1.5 mm grid was close to that of the 2 and 1 mm grids, with a difference of less than 5%. The mesh size was set to 1.5 mm, resulting in 87,818 elements for the cortical bone and 81,725 elements for the cancellous bone. The mesh convergence was found to be less than 5%, confirming the effectiveness of the model.

### Validation of model effectiveness

**FIGURE 3:**
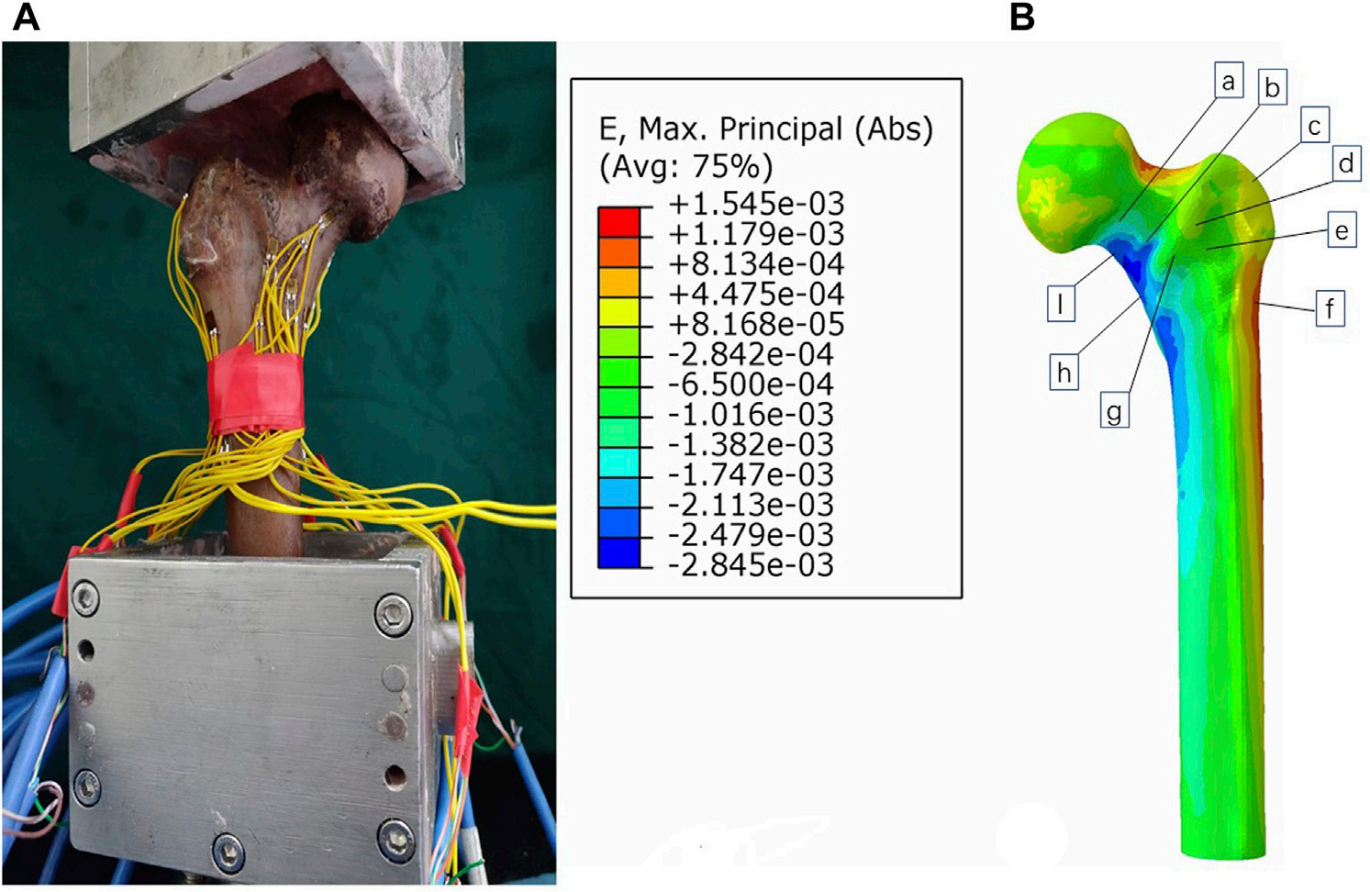
Validation of the model by comparing biomechanical study **(A)** and finite element analysis **(B)**.

**FIGURE 4:**
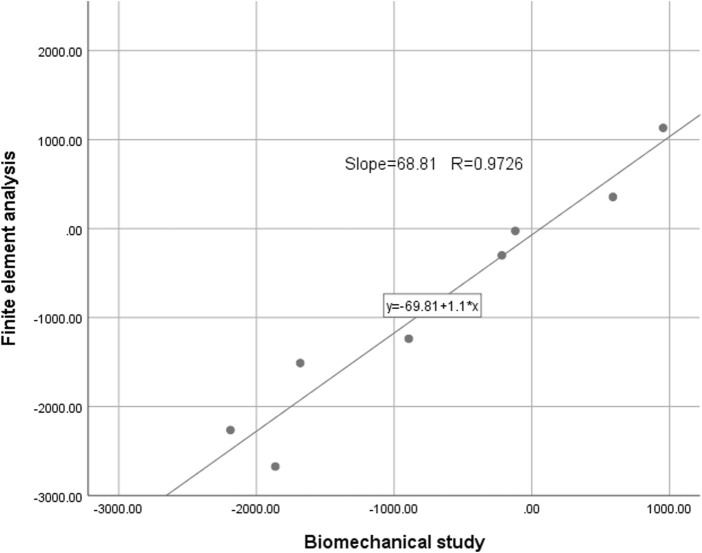
The intraclass correlation coefficient of strain values detected by the two methods was 0.9726 (*p* < 0.01).

In this study, we employed biomechanical analysis to validate the results obtained from finite element analysis. A femoral specimen was screened by X-ray film to exclude other diseases that may affect the bone abnormality. The spatial locations and fixation methods of the proximal femur in the biomechanical study were kept consistent with those in the finite element model, and the nine strain gauges were attached to the surface of the proximal femur corresponding to that in the finite element analysis. To do so, we applied a load of 1,800 N to the proximal femur for both finite element analysis and biomechanical study and recorded strain values at nine marker points. The comparison between the results of the biomechanical study and finite element analysis revealed insignificant differences, affirming the effectiveness of our models (Figures 3, 4). In this study, the stiffness of normal proximal femur was 0.64 KN/mm, which is within the measurement interval reported in the literature [(0.76 ± 0.26) KN/mm] (Papini et al., 2007). The maximum stresses for internal fixation and bone uniaxial of CS fracture fixation model loading were found to be 159.71 and 30.93 MPa, respectively, which is similar to the results (128.77 and 54.62 MPa for internal fixation and bone) of the work of Huang et al. (2023). In addition, the finite element model of the normal proximal femur was validated effectively.

## Results

### Intact proximal femur model

**FIGURE 5:**
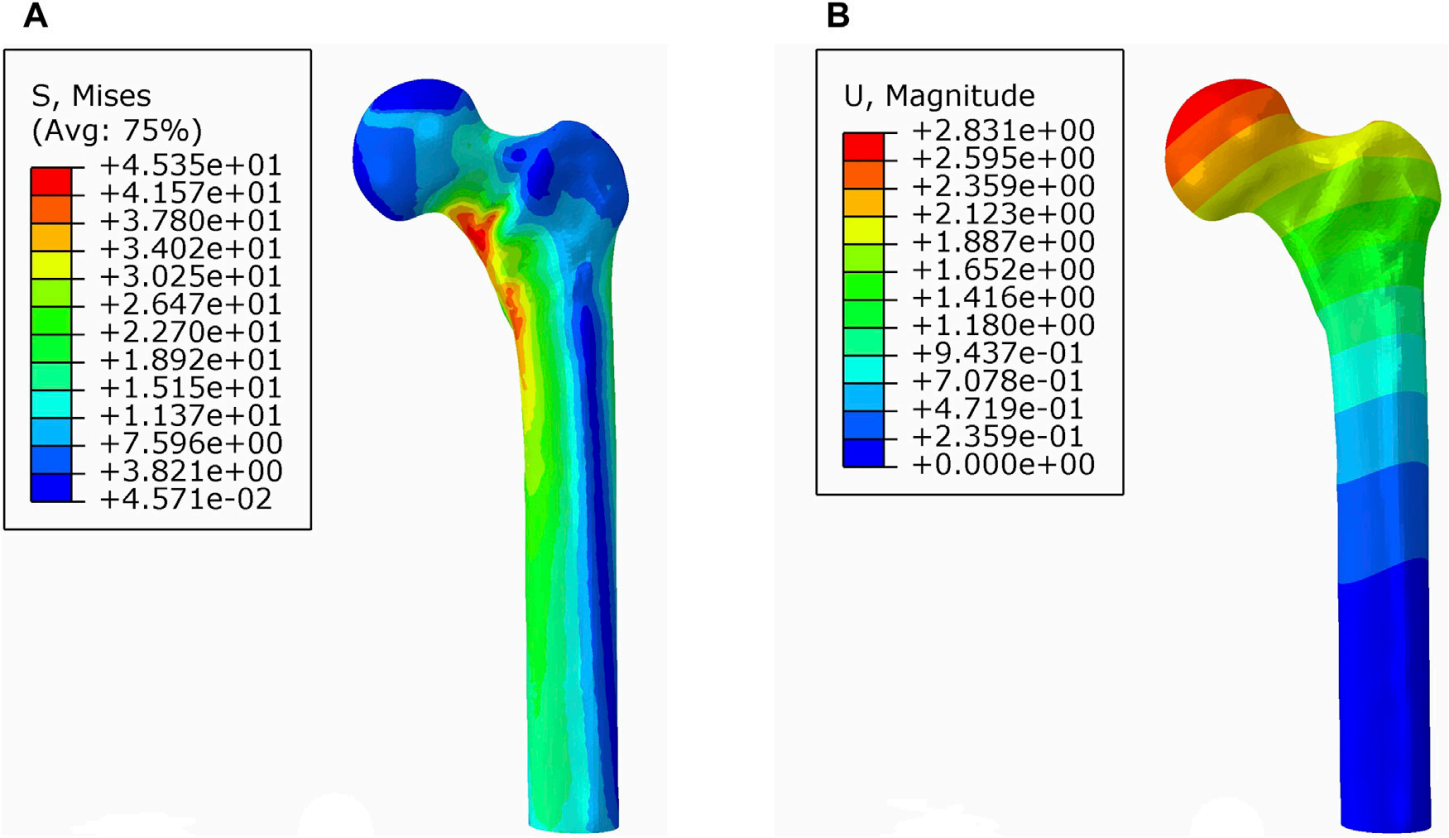
von Mises stress distribution **(A)** and displacement distribution **(B)** of the normal proximal femur.

In the analysis of the intact proximal femur model, the maximum stress and maximum displacement were measured at 45.35 MPa and 2.83 mm under axial load, respectively (Figure 5).

### Stress distribution

**FIGURE 6:**
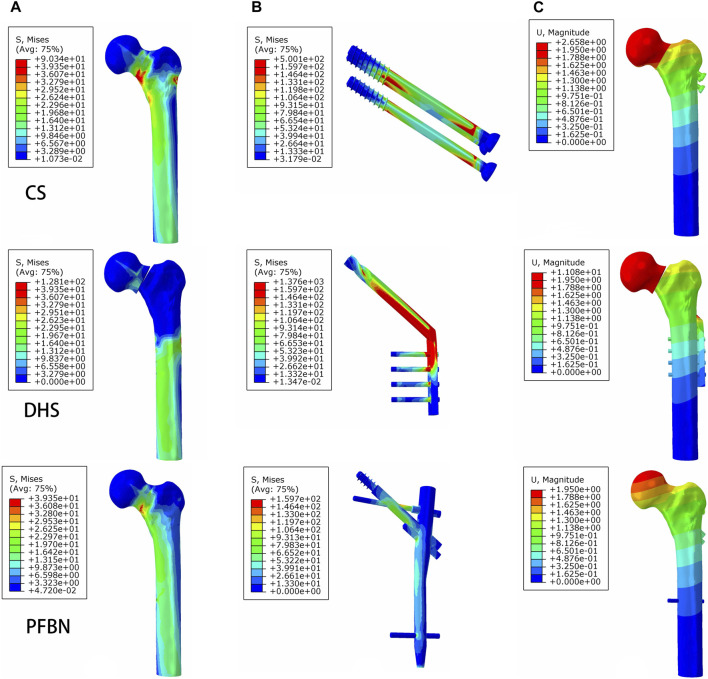
PFBN improved the stress distribution of the proximal femur **(A)** and implant **(B)** and enhanced stability **(C)** under axial load.

**FIGURE 7:**
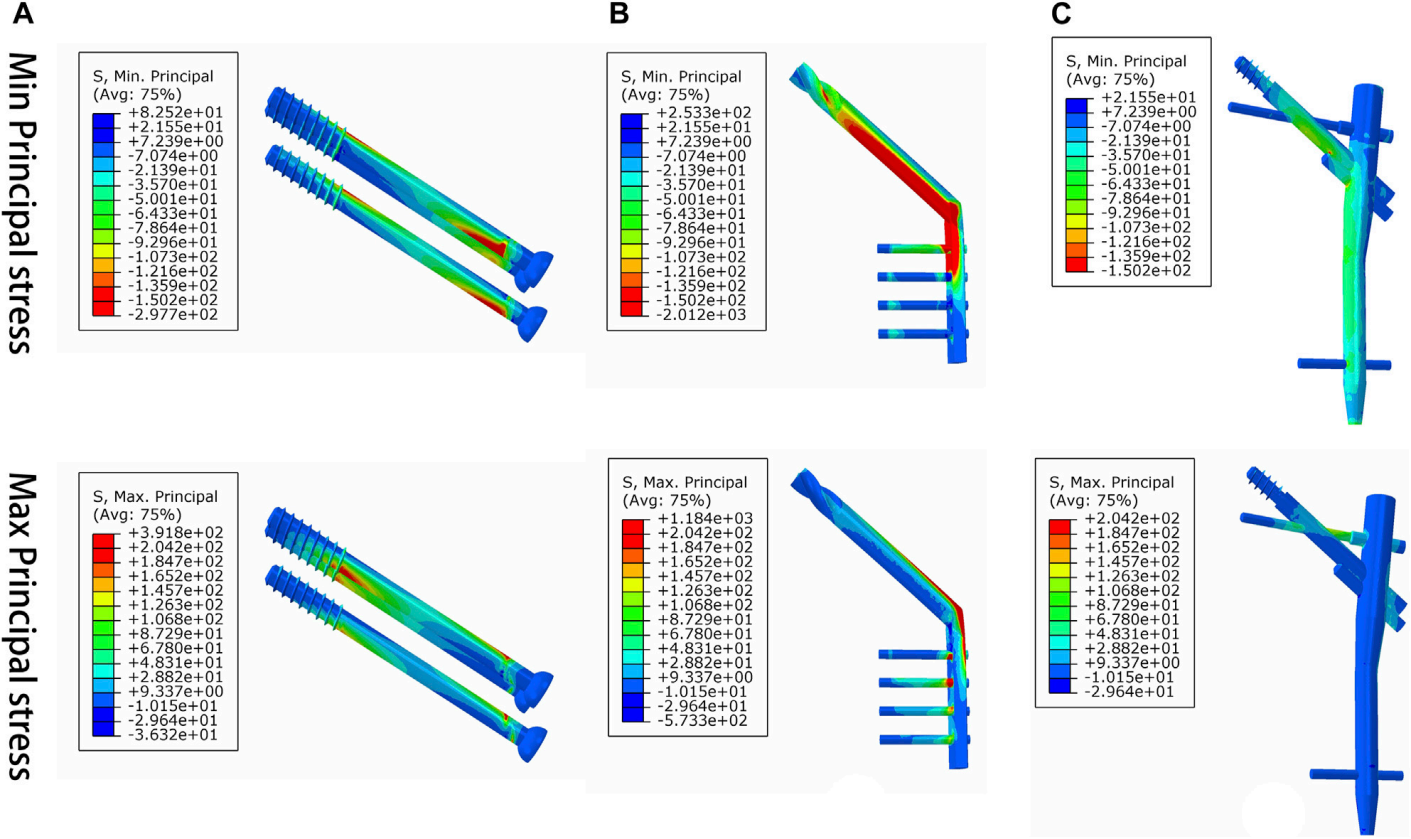
Maximum principal stress and minimum principal stress distribution of three implant models for the treatment of femoral neck fracture. **(A)** CS, **(B)** DHS, and **(C)** PFBN.

Under axial load, we observed the peak von Mises stress, maximum principal stress, and minimum principal stress of the PFBN model. These values were 159.71, 204.23, and −150.18 MPa, respectively. Comparatively, they accounted for 11.61%, 17.25%, and 74.65% of the corresponding values for the DHS, and 31.93%, 52.12%, and 50.45% of those for the CS (Figures 6, 7; Tables 1, 2).

**TABLE 1:** Peak stress and maximum displacement of three implant fixation models for treating femoral neck fracture under axial load.

Implant model	Maximum stress (MPa)		Maximum displacement (mm)	
	Bone	Implant	Fixation model	Relative fracture surface
CS	90.34	500.68	2.66	0.79
DHS	128.13	1376.41	11.08	6.50
PFBN	39.35	159.72	1.95	0.17

**TABLE 2:** Peak values of maximum and minimum principal stress of three implant models for fixing femoral neck fracture (MPa).

Implant model	Max principal stress	Min principal stress
CS	391.81	−297.69
DHS	1184.21	−201.17
PFBN	204.18	−150.23

**FIGURE 8:**
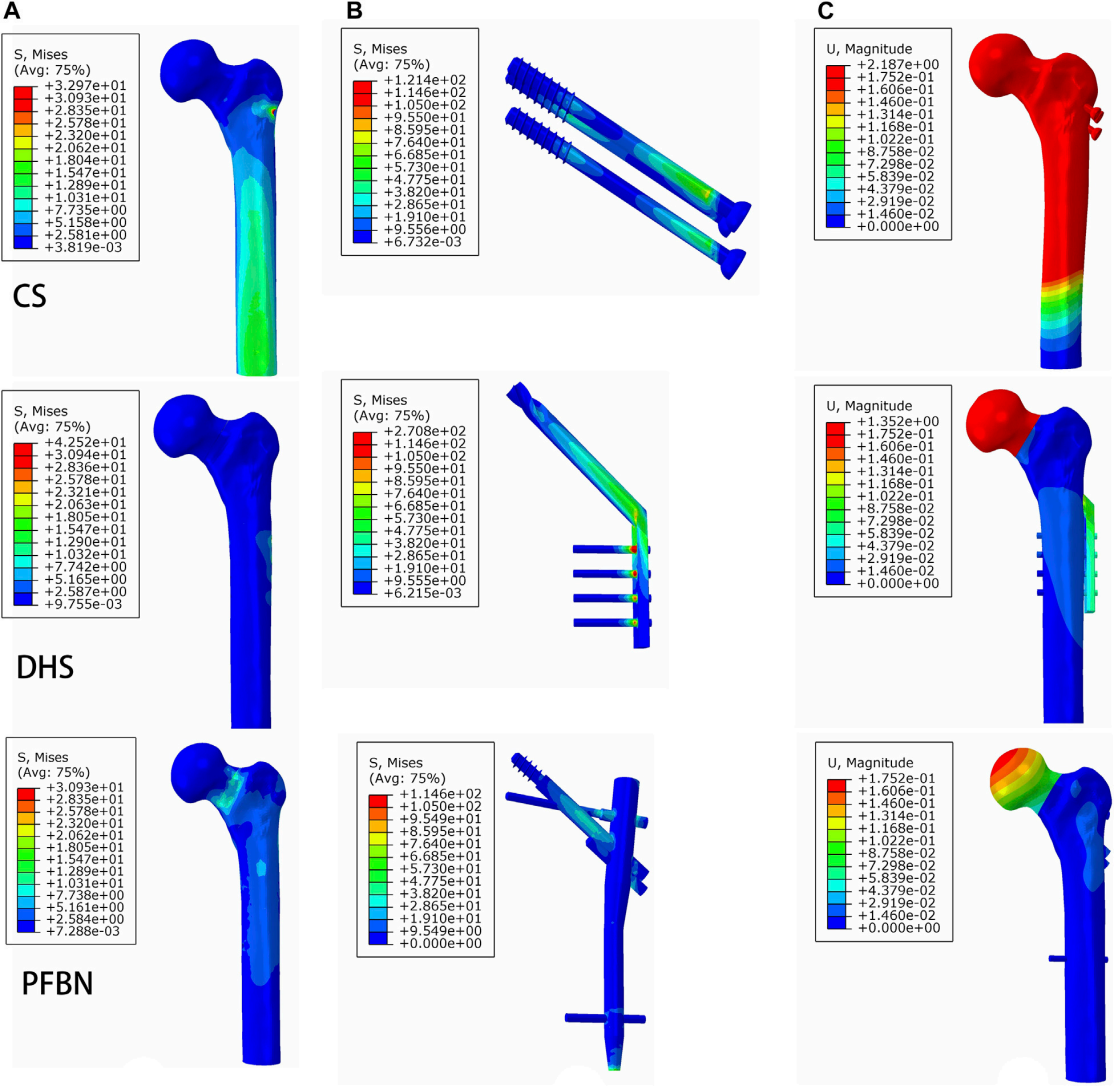
PFBN improved the stress distribution of proximal femur **(A)** and implant **(B)** and enhanced stability **(C)** under bending load.

Additionally, the peak von Mises stress for both bone and internal fixation in the PFBN model was 30.93 and 114.59 MPa under bending load, respectively. These values represented 72.74% and 83.33% of the corresponding values for the DHS and 93.84% and 94.39% of those for the CS (Figure 8; Table 3).

**TABLE 3:** Peak stress and maximum displacement of three implant models for treating femoral neck fracture under bending load.

Implant model	Maximum stress (MPa)		Maximum displacement (mm)
	Bone	Implant	Fixation model
CS	32.97	121.42	2.19
DHS	42.52	270.83	1.35
PFBN	30.93	114.56	1.75

**FIGURE 9:**
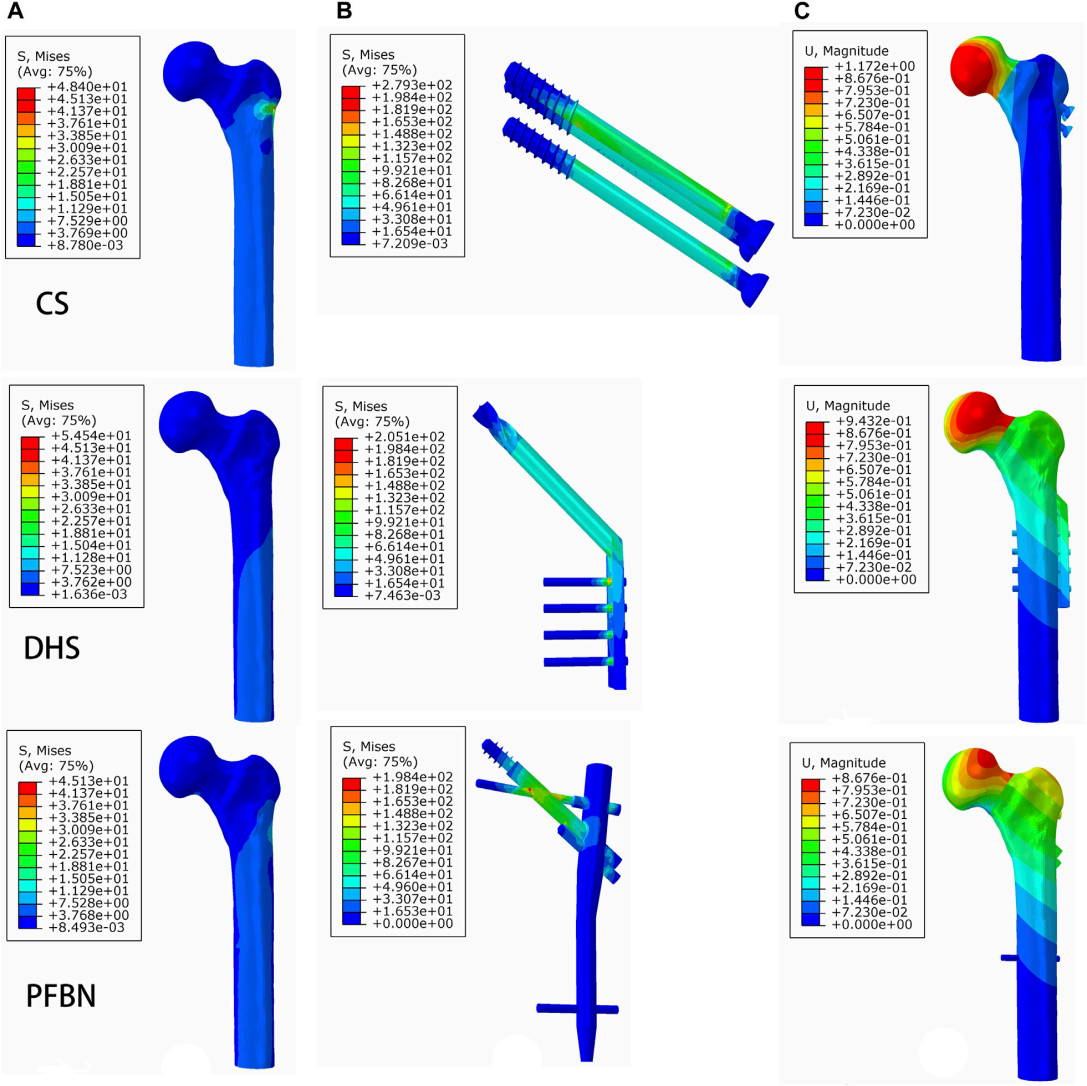
PFBN improved the stress distribution of the proximal femur **(A)** and implant **(B)** and enhanced stability **(C)** under torsion load.

Furthermore, the peak von Mises stress for both bone and internal fixation in the PFBN model was 4.51 and 1.98 MPa under torsion load, respectively. These values accounted for 82.75% and 83.33% of the values in the DHS fixation model and 93.24% and 96.72% of those in the CS fixation model (Figure 9; Table 4).

**TABLE 4:** Peak stress and maximum displacement of three implant models for stabilizing femoral neck fracture under torsion load.

Implant model	Maximum stress (MPa)		Maximum displacement (mm)
	Bone	Implant	Fixation model
CS	4.84	279.34	1.17
DHS	5.45	205.08	0.94
PFBN	4.51	198.36	0.87

### Displacement distribution

Under axial load, we measured the maximum displacement and maximum relative displacement of the fracture sections in the PFBN fixation model at 1.95 and 0.79 mm, respectively. These values were 17.60% and 2.63% of those in the DHS fixation model and 73.58% and 21.44% of those in the CS fixation models (Figure 6; Table 1).

Under bending load, we found that the maximum displacement in the CS and DHS fixation models was 12.48 times and 7.72 times higher, respectively, compared to that in the PFBN fixation model (Figure 8; Table 3).

Finally, the maximum displacement is 1.34 and 1.08 times larger in the CS and DHS models, respectively, than in the PFBN fixed model under torsional load (Figure 9; Table 4).

## Discussion

This study mainly finds that compared to the DHS and CS, the PFBN improves the stress distribution of bone and implants and increases the axial stability and bending strength of the fracture fixation model. In addition, the PFBN exhibits superior biomechanical characteristics in resisting tension and compression forces for the fixation of femoral neck fractures, aligning more closely with the stress conduction of the normal proximal femur. As a result, the PFBN displays significant potential for improving the treatment of femoral neck fractures.

According to our results, the PFBN fixation model has 1.36–5.68 times higher axial stability, 7.7–12.48 times higher bending stability, and 1.08–1.34 times higher torsion stability than the DHS and CS fixation models. In addition, the stress extremes of bone and implant in the PFBN fixation model range from 11.61% to 96.72% of those in the DHS and CS fixation models under three loading conditions. The innovative crossed structure of the PFBN serves to diminish the stress concentration of screws, cortical bone, and cancellous bone, helping to reduce the risk of shortening of the femoral neck, necrosis of the femoral head, and backout (Stoffel et al., 2017; Xu et al., 2022). In addition, the maximum relative displacement of the fracture section in the PFBN is lower than that of the CS and DHS under axial loading. It is expected to reduce the risk of necrosis of the femoral head and fracture non-union caused by the displaced fracture surface. Therefore, the PFBN has superior resistance to axial loads, bending loads, and torsional loads than the CS and DHS for the treatment of femoral neck fracture.

There are several structural features of the PFBN that explain the biomechanical differences between the PFBN and CS/DHS. First, unlike the single fixation screw of the DHS and the three parallel screw structure of the CS, the crossed structure of the PFBN forms a stable integrity with the bone, thus increasing the holding force on the proximal fracture fragment and enhancing the stability of the fracture fixation model. This was also verified by the stress distribution of implants and bones. The DHS and CS lead to great movement of the proximal fracture section for stabilizing femoral neck fracture, suggesting that the single and multi-parallel screw structure had insufficient holding power resulting in drawbacks such as femoral head varus collapse and non-union. For treating femoral neck fractures, non-parallel screw fixation techniques such as the biplane double-support screw fixation, proximal femoral plate, and femoral neck system can offer greater angular stability (Stoffel et al., 2017; Viberg et al., 2017; Augat et al., 2019). Consequently, the maximum relative displacement of fracture sections in the PFBN fixation model is less than that in the CS and DHS fixation models. Moreover, the cross structure of the PFBN allows for a more efficient transfer of compression and tension forces than the CS and DHS, reducing stress concentration in individual screws. As our results show, the maximum principal stress of the PFBN is 17.25% of the DHS and 52.12% of the CS, while the minimum principal stress is 74.65% of the DHS and 50.45% of the CS. The cross structure is designed to facilitate the transfer of compression and tension forces between screws, thereby reducing stress concentration on individual screws and mitigating postoperative complications related to proximal femoral plates (Augat et al., 2019; Xu et al., 2022). Finally, the PFBN is a centrally fixed internal fixation, which reduces the lever arm and stress concentration of the implant. Numerous studies have demonstrated that intramedullary fixation is highly effective in providing excellent biomechanical characteristics and clinical outcomes for femoral neck fractures (Augat et al., 2019; Huang et al., 2023). Based on our analysis, the PFBN is a more suitable treatment option for femoral neck fractures than the CS and DHS.

In clinical practice, the DHS and CS are the most commonly employed internal fixation methods for femoral neck fractures (Florschutz et al., 2015). Both of these methods are grounded in the anterograde compression theory, aiming to convert joint forces into compressive forces on the fracture surfaces in the direction of the fixation screw (Augat et al., 2005; Augat et al., 2019; Nan et al., 2023). Attempts to enhance internal fixation have involved increasing the number of screws and adding a medial femoral plate (Zhan et al., 2020; Zelle et al., 2022). However, these modifications did not adequately address tension resistance, resulting in only marginal reductions in complications. The non-parallel screw structure of the proximal femoral locking plate can potentially offer enhanced overall stability (Aminian et al., 2007; Nowotarski et al., 2012). Nonetheless, this non-parallel configuration can lead to stress concentration in individual screws, resulting in screw backout and loosening (Schneider et al., 2015; Stoffel et al., 2017). In response to these challenges, our research team pioneered the concept of TSF, for which we obtained six patents. The support and tension screws form a mixed triangle with the cortical bone at the femoral head and the adjacent cancellous bone. Furthermore, the support screw passes through this hole to form a stable triangular structure (metal triangle) with the tension screw and the proximal main nail, effectively reducing the lever arm and stress concentration on the bone (Ding et al., 2022a; Ding et al., 2022b). As eccentric internal fixation, the fixation screws of the CS and DHS are often responsible for tension and compression conduction, which leads to stress concentration and macrostrain of the screw/cortical bone. The cross structure of the PFBN obviously changes the direction of stress transmission, which makes the fixation screws and support screws transmit compression and tension force, respectively. The PFBN is in line with the normal biomechanical characteristics of the proximal femur, which reduces the stress concentration.

Therefore, the PFBN enhances the transmission of tension and compression forces in the femoral neck by mimicking the trabecular structure, closely resembling the natural stress transmission pattern in the proximal femur (Ding et al., 2022a; Wang et al., 2022a; Ding et al., 2022b; Wang et al., 2022b). In addition, the PFBN as well as TSPF also reduce stress concentrations, improve stress conduction, and enhance fracture stability in the fixation of intertrochanteric fractures, demonstrating excellent results in clinical applications (Ding et al., 2022a; Wang et al., 2022a; Ding et al., 2022b; Wang et al., 2022b; Zhao et al., 2023). This provides a solid foundation for the clinical treatment and promotion of femoral neck fractures.

Currently, there are two methods including biomechanical study and finite element analysis for orthopedics research methods. FEA is a numerical model that can accurately predict biomechanical characteristics such as orthopedic stability and risk of internal fixation failure. In addition, finite element analysis is non-invasive and simple to use, making it widely used in orthopedic biomechanics research. This study analyzes the characteristics of stress distribution and displacement distribution in bones and implants using finite element analysis, which well demonstrates the role of the PFBN in the stress transmission of stabilizing femoral neck fracture. Moreover, the fracture fixation stability was assessed using the three most common static loading conditions, including axial, torsional, and bending loads during hip activity. In this study, the maximum loads for three types of motion during normal hip joint activity were set to 1,800 N (three times the body load), 15 nm, and 150 N. These parameters can effectively assess the biomechanical state with the greatest risk of internal fixation failure, making them sufficient for stability assessment. In addition, the static load was used to evaluate fracture fixation stability, which is also a common method in finite element analysis (Li et al., 2019; Zhan et al., 2020; Huang et al., 2023).

Our study is not without limitations. First, the assignment of isotropic and linear elastic properties to the cortical and cancellous bone in this study does not entirely align with the actual material properties of bones. Second, the finite element analysis showed the biomechanical characteristics of three fixation models but ignored changes in bone mass, soft tissue, and blood vessels after fracture fixation, which affect the prognosis of intertrochanteric fractures. We have added it to the limitations of the study. Third, the selection of CT images of healthy adults for constructing 3D models is currently a common approach for finite element analysis (Li et al., 2019; Zhan et al., 2020; Huang et al., 2023). However, the representativeness of finite element analysis based on CT data was limited because the methods ignored some factors including BMI, obesity, and muscle atrophy that may have influenced the effectiveness and reliability of the results (Chen et al., 2022). Finally, although the PFBN displays excellent biomechanical properties, additional clinical trials are necessary to confirm the clinical value of the PFBN. There is a lack of clinical evidence assessing the ease of surgery, operation time, bleeding, and recovery time of the PFBN and DHS/CS for the treatment of femoral neck fractures.

In summary, when compared to the DHS and CS, the PFBN demonstrates improvements in stress concentration, stress propagation, and overall stability in the femoral neck fracture fixation model. These improvements better match the tissue structure and biomechanical properties of the proximal femur. As a result, the PFBN exhibits significant potential for the clinical treatment of femoral neck fractures.

## Data Availability

The original contributions presented in the study are included in the article/supplementary material; further inquiries can be directed to the corresponding authors.
